# Case Report: The effect of early initiation of eteplirsen treatment on the cardiac and motor disease course in an individual with Duchenne muscular dystrophy

**DOI:** 10.3389/fped.2026.1672692

**Published:** 2026-07-09

**Authors:** Yamen Masalha, Yoav Zehavi, Banan Musallam, Majd Khamaysi, Maali Abu Omer, Daniel L. Fink, Ronen Spiegel

**Affiliations:** 1 Pediatric Department B, Emek Medical Center, Afula, Israel; 2 Rappaport School of Medicine, Technion, Haifa, Israel; 3 Pediatric Neurology Unit, Emek Medical Center, Afula, Israel; 4 Pediatric Cardiology Unit, Emek Medical Center, Afula, Israel

**Keywords:** cardiomyopathy, case report, Duchenne muscular dystrophy, eteplirsen, exon skipping therapy

## Abstract

Duchenne muscular dystrophy (DMD) is an X-linked disorder characterized by progressive muscle weakness. Here we provide a case report for a male aged 7 years and 3 months diagnosed with DMD after detecting a deletion of exons 48–50 of the *DMD* gene. The child initially presented with elevated transaminases during a febrile illness at 17 months. Further evaluation revealed significantly increased creatine phosphokinase and troponin levels. He exhibited delayed gross motor milestones and developed left ventricular diastolic dysfunction. At 24 months of age, weekly intravenous eteplirsen therapy was initiated, along with enalapril to manage cardiac symptoms. Over 5 years, the patient demonstrated stable cardiac function and gradual motor improvement. Despite persistent hyperlordosis and waddling gait, his 6-minute walk test remained stable. To our knowledge, this is the first documented case of DMD reporting cardiac outcomes after eteplirsen treatment in a patient as young as 24 months of age.

## 1 Introduction

Duchenne muscular dystrophy (DMD) is a rare, X-linked degenerative, neuromuscular disease estimated to affect 1 in 5,000 births worldwide (1). DMD is caused by mutations in the *DMD* gene, resulting in the absence of functional dystrophin, a critical protein for muscle integrity (2). This absence leads to continuous muscle damage, motor deterioration, loss of ambulation, and eventual respiratory impairment and cardiomyopathy (1–4).

DMD is generally characterized by gradual motor impairment, with symptoms typically appearing in early childhood, including delayed motor milestones, muscle weakness, and specific motor deficits such as Gower's sign (2). Cardiac complications are often a significant concern in the later stages of DMD disease progression (5–7).

Cardiomyopathy, particularly left ventricular dysfunction, is a major contributor to morbidity and mortality in individuals with DMD, usually emerging in the teenage years (5, 7). Early-onset cardiomyopathy is an uncommon and rare manifestation of DMD in infants and very young children (2, 8).

While there is no cure for DMD, current treatments and standard of care aim to slow disease progression, improve quality of life, and manage symptoms (9). Glucocorticoids are the primary standard of care and help maintain muscle strength, and prolong ambulation, although long-term use can lead to side effects (10). Emerging treatments, such as gene therapy and exon-skipping agents, target the underlying cause of DMD, enabling dystrophin production and stabilization of muscle function (9).

Eteplirsen is an anti-sense oligonucleotide that is approved by the US Food and Drug Administration to treat individuals with a confirmed mutation of the *DMD* gene that is amenable to exon 51 skipping; namely deletions in the *DMD* gene ending at exon 50 and starting at exon 52 (11, 12). Eteplirsen is designed to bind (or hybridize) to exon 51 of the *DMD* gene, causing this section to be skipped during splicing. Restoration of the translational reading frame leads to the production of a shortened but functional dystrophin protein (13, 14).

Here we present a case report demonstrating the long-term effects of early eteplirsen treatment on the stabilization of motor function and cardiac outcomes in a child with DMD showing very early-onset cardiomyopathy.

## 2 Case study

### 2.1 Initial presentation and treatment initiation

A male patient, aged 7 years and 3 months, initially presented with elevated transaminases during a febrile viral illness at 17 months of age. The patient is the fourth child of healthy parents and there is no heredity or medical history within the family. The patient's growth and development were generally unremarkable, although his motor milestones were mildly delayed, with crawling observed at 13 months of age, assisted walking seen at 17 months of age, and walking independently commencing at 19 months of age.

Despite recovery from the viral illness within days, the elevation in transaminases persisted, prompting further investigation, which detected a serum creatine phosphokinase level of 15,000 IU/L (normal range: 20–180 IU/L) and elevated troponin levels (122 ng/L; normal range: 0–18 ng/L).

Physical examination revealed a wide-based gait, pseudohypertrophy of the calves, decreased patellar and ankle reflexes, and cardiac auscultation indicating a loud fourth heart sound (S4) with a gallop rhythm, which are indicative of DMD clinical phenotypes.

An echocardiography confirmed left ventricular diastolic dysfunction, and treatment with enalapril (0.35 mg/kg/day) was initiated when the child was 1 year and 9 months of age.

**Figure 1:**
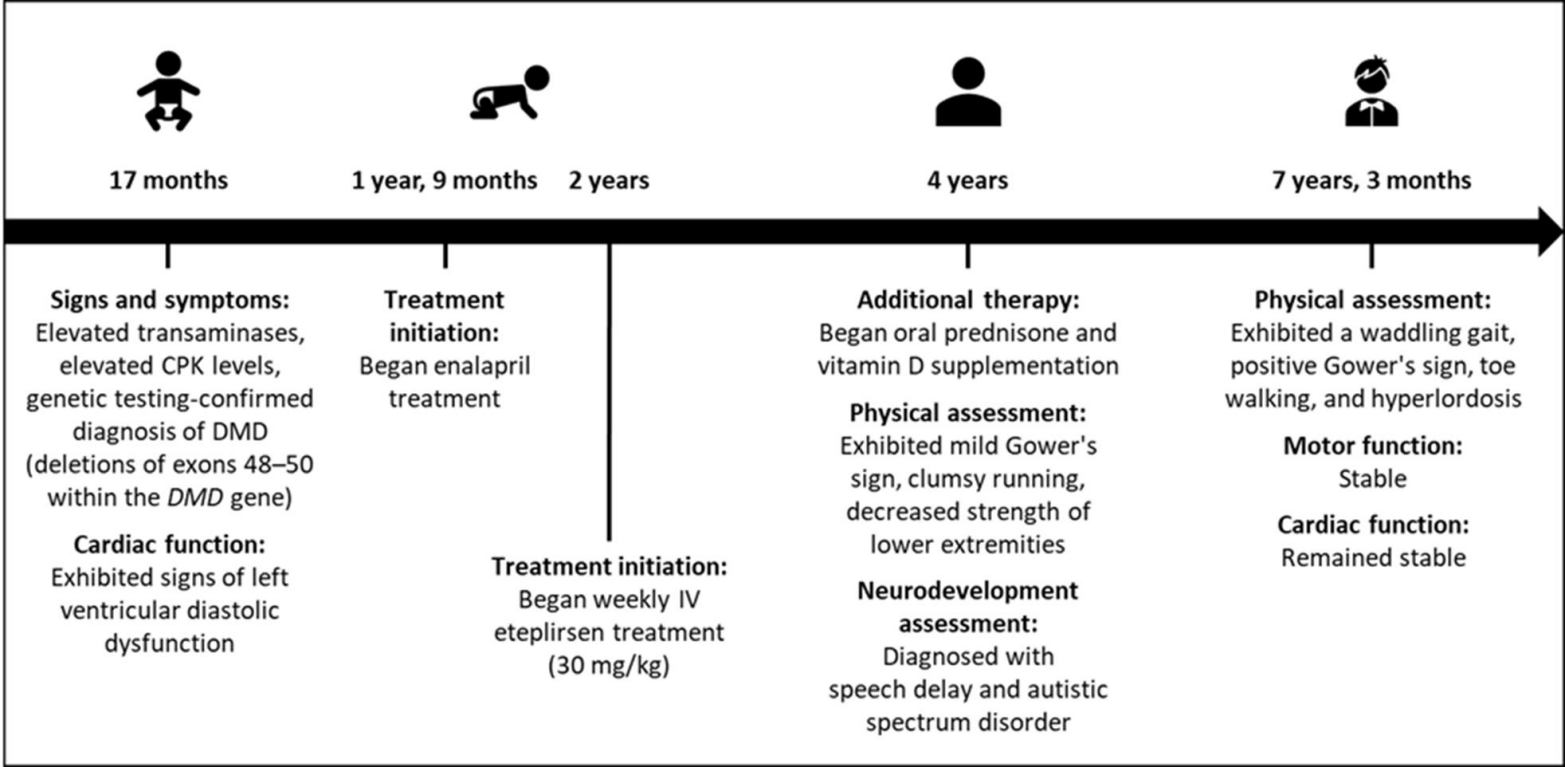
Overview and timeline of the patient case study. CPK, creatine phosphokinase; DMD, Duchenne muscular dystrophy; IV, intravenous.

To further investigate the clinical suspicion of DMD, blood samples were obtained for genetic analysis. Genetic testing confirmed a deletion of exons 48–50 within the *DMD* gene, establishing the diagnosis of DMD (Figure 1).

### 2.2 Therapeutic management and assessments

Given that the patient had an identified mutation in the *DMD* gene that was amenable to exon 51 skipping therapy, eteplirsen treatment was initiated when the patient was 24 months of age. Eteplirsen was administered at a dose of 30 mg/kg weekly via intravenous infusion. Over the 5 years of receiving eteplirsen, the treatment was well tolerated, with no significant complications or adverse events reported. Treatment tolerability was assessed through routine clinical evaluations conducted at regular follow-up visits, including monitoring for adverse events, review of laboratory test results, functional assessments and parental reporting of symptoms or behavioral changes.

The intensity of S4 decreased after initiation of enalapril and then completely disappeared at 2.2 years when the patient received enalapril and eteplirsen. The enalapril dose was last increased to 0.9 mg/kg/day at 4.5 years of age and has remained unchanged. This increase was undertaken due to reappearance of early cardiac dysfunction on the lower prophylactic dose of enalapril with return of the abnormal S4 and E wave-to-A wave (E/A) ratio. Additionally, oral prednisone and vitamin D supplementation were introduced when the patient was 4 years of age to support muscle strength and skeletal health.

The patient and his family have demonstrated excellent compliance with the therapeutic plan. The patient is monitored regularly with motor evaluations and comprehensive neurologic assessments are undertaken along with cardiovascular examinations, including electrocardiograms and echocardiograms conducted by a pediatric cardiologist.

### 2.3 Cognitive and motor development

At 4 years of age, the patient was diagnosed with a speech delay and autism spectrum disorder, though formal hearing assessments were normal. Muscle weakness was observed, along with Gower's sign and clumsy running. At this time, the distance reached on the 6-minute walk test (6MWT) was 198 meters.

Over the next 2 years, the patient showed some improvements in motor function although significantly below age matched peers, reaching a peak 6MWT distance of 280 meters at 6 years of age. The patient was able to climb 5–10 stairs without support. Orthopedic measures, including the use of ankle braces, were introduced to preserve his range of motion.

**Figure 2:**
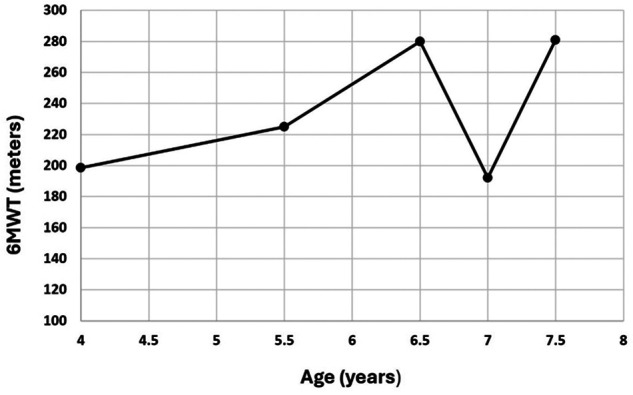
6MWT showed stabilization of exercise tolerance as the child aged. 6MWT, 6 -minute walk test.

At the most recent assessment, when the patient was aged 7 years and 3 months, he demonstrated stable motor function, although he continued to exhibit a waddling gait, positive Gower's sign, toe walking, and hyperlordosis. The patient could climb five stairs with some assistance, run a short distance of 10 meters, and achieved a 6MWT distance of 281 meters (Figure 2). The patient's North Star Ambulatory Assessment total score remained stable at 13 points. Fine motor skills, such as writing and dressing, showed improvement, although the patient still required some assistance. Overall, the patient's motor development was delayed compared with age-matched boys; however, the expected motor regression has been suspended.

### 2.4 Cardiac function

At the initial DMD diagnosis, the patient presented with signs of left ventricular diastolic dysfunction, along with elevated serum high-sensitivity troponin T levels, both of which indicated early-onset cardiomyopathy.

Over the 5 years following initiation of eteplirsen and enalapril treatment, repeated assessments of serum troponin T levels showed stable elevations with no further increases. The patient's cardiac status remained stable, with no significant progression of cardiomyopathy (Table 1). As mentioned, enalapril dosage was increased from the lower prophylactic dose to a higher dosage (0.9 mg/kg/day) at 4.5 years due to the reoccurrence of early cardiac dysfunction around 3.5–4 years of age (Table 1). This increase was tolerable and not associated with any side effects.

**Table 1:** Cardiac and echocardiogram parameters showed stable cardiac function with slight clinical improvement as the child aged.

Age (years)	Enalapril dose (mg/kg/day)	S4 intensity	E/A ratio	SV (mL)	EF (%)	FS (%)	PW (mm)	IVS (mm)	LVESD (mm)	LVEDD (mm)	Z-score	ECG findings
1.6	–	3+	1.12	26	77	44	0.5	0.5	17	30	0.3	BVH
1.9	0.35	2+	1.32	19	66	35	0.5	0.5	18	28	−1.4	BVH
1.11	0.35	1+	1.11	12	76	42	0.5	0.5	13	22	−4.4	BVH
2.2^a^	0.35	0	1.79	25	68	37	0.5	0.5	19	30	−0.74	BVH
2.8	0.4	0	1.02	24	75	42	0.5	0.5	17	29	−1.69	BVH
2.11	0.4	0	1.64	26	63	33	0.5	0.5	22	32	−0.2	BVH
3.6	0.3	1+	0.92	20	60	35	0.5	0.5	20	29	−2.2	BVH
5.3	0.9	1+	1.55	42	71	40	0.5	0.5	22	37	0.7	BVH
6.2	0.8	0	1.66	38	69	38	0.6	0.6	20	33	−1.36	BVH

BVH, biventricular hypertrophy; E/A ratio, E-wave and A-wave ratio (early to late ratio); ECG, electrocardiogram; EF, ejection fraction; FS, fractional shortening; IVS, interventricular septum; LVEDD, left ventricular end-diastolic diameter; LVESD, left ventricular end-systolic diameter; PW, posterior wall; S4, fourth heart sound; SV, stroke volume.

a Patient started eteplirsen (30 mg/kg) treatment at 24 months of age.

Echocardiogram assessments showed that key markers of cardiac function, such as ejection fraction, stroke volume, and E/A ratio remained within stable ranges. Physical examinations revealed the disappearance of the fourth heart sound (S4) suggesting improvements in cardiac function and allowed mild decrease in enalapril treatment to (0.8 mg/kg/day). Tissue Doppler and E/E′ ratio measurements were unavailable due to technical limitations. Cardiac MRI was not performed because it would have required anesthesia, which was not considered unwarranted in this patient with neuromuscular disease.

## 3 Discussion

This case study highlights the potential benefits of initiating eteplirsen therapy early in the disease course in a patient with DMD showing signs of early-onset cardiomyopathy. The patient was diagnosed with DMD following the detection of a deletion of exons 48–50 within the *DMD* gene. The patient subsequently began treatment with enalapril at 21 months of age and eteplirsen at 24 months of age. Enalapril is an angiotensin-converting enzyme inhibitor. It exerts its beneficial effects in cardiomyopathy through blockade of the renin-angiotensin aldosteron system which prevents the conversion of angiotensin I to angiotensin II and reduces aldosterone secretion thereby reversing adverse cardiac remodeling and improving myocardial function (15).

In addition to early motor and cardiac challenges, the patient also presented with speech delay and symptoms consistent with autism spectrum disorder, which are commonly observed in individuals with DMD (2). Despite the severe DMD presentation, the patient demonstrated stable motor function and cardiac performance over 5 years of treatment, providing valuable insights into the long-term effects of exon-skipping therapy. The patient remained highly compliant throughout treatment and experienced no clinically relevant adverse events.

The findings from this case report suggest that early intervention with eteplirsen therapy may be able to delay the progression of motor deterioration, enabling individuals with DMD to retain functional independence for longer. In this case study, the patient's consistent motor stabilization suggests that early eteplirsen therapy may extend ambulation and mitigate the rapid decline of motor function typically seen in DMD.

Previous studies have suggested that exon-skipping therapies may stabilize motor function by increasing dystrophin production, and this case study adds to the growing body of evidence supporting early treatment of individuals with DMD who have mutations in the *DMD* gene amendable to exon-skipping treatment (4, 11, 16). Notably, the patient in this case study showed stabilization of performance on the 6MWT. Similarly, a 3-year study involving 12 individuals with DMD reported that those receiving eteplirsen demonstrated better performance on the 6MWT and a slower decline in ambulatory function compared with matched historical cohorts (14).

Cardiomyopathy is a leading cause of morbidity and mortality in individuals with DMD (5, 7); early diagnosis and stabilization of heart function are critical for improving life expectancy (5). The natural history of cardiomyopathy in individuals with DMD is characterized by cardiac fibrosis, arrhythmias (primarily supraventricular), and heart failure, finally culminating in ventricular dysfunction and ventricular dilatation (5, 17). Myocardial damage at a cellular level occurs before the onset of clinically evident cardiac dysfunction in DMD (18).

While cardiomyopathy can develop at any age in individuals with DMD, preclinical cardiac involvement has been identified in 25% of children under 6 years of age, increasing to 60% among children aged 6–10 years of age, before declining in prevalence with age (19). Nigro et al. reported that clinically apparent cardiomyopathy typically becomes evident after 10 years of age, often presenting with electrocardiogram abnormalities and sinus tachycardia (18, 20). However, other studies have reported that cardiomyopathy usually manifests around 14–15 years of age and is highly prevalent in individuals over 18 years of age (21, 22).

It is therefore uncommon for a patient with DMD to present with heart issues as early as 17 months of age, as seen in this case study. This early-onset cardiac involvement highlights the need for proactive cardiac monitoring and intervention as needed in DMD, even in very young patients. As motor outcomes are often the primary focus in the management of DMD, it is important to consider and address any cardiac involvement early in the disease course.

Cardiac problems in individuals with DMD often go undetected without detailed examination, as many individuals show no classic symptoms of heart failure or the symptoms may go unrecognized (22, 23). Since most individuals with DMD are wheelchair bound and do not engage in activities causing increased cardiac output, any cardiac issues they have often remain unnoticed, leading to delays in proper evaluation and the late initiation of pharmacologic intervention (23).

In this case study, stabilization of cardiac function with no further deterioration was observed following the initiation of treatment with eteplirsen and enalapril. Previous studies have already shown a protective role for angiotensin-converting enzyme inhibitors, such as enalapril, in the progression of DMD-related cardiomyopathy by inhibition of the cardiac remodelling mechanism (24, 25). However, data assessing the effect of eteplirsen on the cardiac disease course in DMD are limited. The potential cardiac benefits observed with eteplirsen in this case study align with findings from two other recent studies, which suggest that exon-skipping therapies may contribute to maintaining, and preventing deterioration of, cardiac function in DMD (26, 27). Iff et al. reported that among patients with DMD due to exon 51 amenable mutations, eteplirsen treatment was associated with a significantly decreased decline in left ventricular ejection fraction in agreement with better preservation of cardiac function compared with a matched control group of patients with DMD not receiving eteplirsen (27).

Additionally, studies specifically assessing the natural progression of infantile-onset cardiomyopathy in individuals with DMD treated with eteplirsen remain limited. To our knowledge, this study is the first to report cardiac outcomes in a patient with DMD as young as 17 months of age. These findings support the recommendation to initiate treatment early, before signs of ventricular dysfunction are detectable, to prevent or delay the early onset of cardiomyopathy and heart failure (23), in addition to the known motor effect of slowing disease progression and preservation of independent ambulation.

It is important to acknowledge that this case study focuses on a single patient, which limits the generalizability of the findings to a broader population of individuals with DMD. As a result, this study cannot provide comprehensive insights into how other individuals with DMD might respond to eteplirsen treatment or whether others may experience a similar clinical disease course. Further prospective research involving larger cohorts, especially among patients starting treatment in infancy or early childhood, is required to confirm these findings and better understand the potential benefits of exon-skipping therapies on cardiac and motor outcomes in individuals with DMD.

## 4 Conclusion

Eteplirsen is a novel disease-modifying treatment for individuals with DMD who have mutations amenable to exon 51 skipping. This case study highlights the potential for early intervention with eteplirsen and the impact that long-term combined treatment with eteplirsen and enalapril by two separate mechanisms of action may have on the overall disease progression including motor function and cardiac outcomes, even when early-onset cardiomyopathy is present. While these findings are promising, larger studies or case series are necessary to confirm the long-term cardiac benefits of eteplirsen and to further explore its potential in managing DMD-related cardiomyopathy.

## Data Availability

The original contributions presented as part of this case report are included in the article/Supplementary Material, further inquiries can be directed to the corresponding author.

## References

[1] Asher D Thapa K Dharia S Khan N Potter R Rodino-Klapac L. Clinical development on the frontier: gene therapy for Duchenne muscular dystrophy. Expert Opin Biol Ther. (2020) 20(3):253–74. 10.1080/14712598.2020.1725469 32031420

[2] Duan D Goemans N Takeda S Mercuri E Aartsma-Rus A. Duchenne muscular dystrophy. Nat Rev Dis Primers. (2021) 7(1):13. 10.1038/s41572-021-00248-3 33602943 PMC10557455

[3] Mendell J Sahenk Z Lehman K Nease C Lowes LP Miller NF. Assessment of systemic delivery of rAAVrh74.MHCK7.micro-dystrophin in children with Duchenne muscular dystrophy: a nonrandomized controlled trial. JAMA Neurol. (2020) 77(9):1121–31. 10.1001/jamaneurol.2020.1484 PMC7296461 32539076

[4] Iff J Zhong Y Tuttle E Gupta D Paul X Erik H. Real-world evidence of eteplirsen treatment effects in patients with Duchenne muscular dystrophy in the USA. J Comp Eff Res. (2023) 12(9):e230086. 10.57264/cer-2023-0086 37610303 PMC10690424

[5] Schultz TI Raucci FJ Salloum J Salloum FN. Cardiovascular disease in Duchenne muscular dystrophy: overview and insight into novel therapeutic targets. JACC Basic to Translational Science. (2022) 7(6):608–25. 10.1016/j.jacbts.2021.11.004 35818510 PMC9270569

[6] McNally E Kaltman J Benson W Canter C Cripe L Duan D. Contemporary cardiac issues in Duchenne muscular dystrophy. Circulation. (2015) 131:1590–8. 10.1161/CIRCULATIONAHA.114.015151 25940966 PMC4573596

[7] Lechner A Herzig JJ Kientsch JG Kohler M Bloch KE Ulrich S. Cardiomyopathy as cause of death in duchenne muscular dystrophy: a longitudinal observational study. ERJ Open Res. (2023) 9(5):00176–2023. 10.1183/23120541.00176-2023 37727676 PMC10505954

[8] McNally EM. Cardiomyopathy in muscular dystrophy: when to treat? JAMA Cardiol. (2017) 2(2):199. 10.1001/jamacardio.2016.4910 27926775 PMC5510024

[9] Patterson G Conner H Groneman M Blavo C Parmar MS. Duchenne muscular dystrophy: current treatment and emerging exon skipping and gene therapy approach. Eur J Pharmacol. (2023) 947:175675. 10.1016/j.ejphar.2023.175675 36963652

[10] McDonald CM Henricson EK Abresch RT Duong T Joyce NC Hu F. Long-term effects of glucocorticoids on function, quality of life, and survival in patients with Duchenne muscular dystrophy: a prospective cohort study. Lancet. (2018) 391(10119):451–61. 10.1016/S0140-6736(17)32160-8 29174484

[11] Lim KR Maruyama R Yokota T. Eteplirsen in the treatment of Duchenne muscular dystrophy. Drug Des Devel Ther. (2017) 11:533–45. 10.2147/DDDT.S97635 28280301 PMC5338848

[12] McNally EM Wyatt EJ. Mutation-based therapy for Duchenne muscular dystrophy: antisense treatment arrives in the clinic. Circulation. (2017) 136(11):979–81. 10.1161/circulationaha.117.028382 28893959 PMC5657589

[13] McDonald CM Shieh PB HZ A-H Connolly AM Ciafaloni E Wagner KR. Open-label evaluation of eteplirsen in patients with Duchenne muscular dystrophy amenable to exon 51 skipping. PROMOVI Trial. J Neuromuscul Dis. (2021) 8(6):989–1001. 10.3233/jnd-210643 34120909 PMC8673535

[14] Mendell JR Goemans N Lowes LP Alfano LN Berry K Shao J. Longitudinal effect of eteplirsen versus historical control on ambulation in Duchenne muscular dystrophy. Ann Neurol. (2016) 79(2):257–71. 10.1002/ana.24555 26573217 PMC5064753

[15] Faruqi A Patel P Jain A. Enalapril. ed. StatPearls. Treasure Island (FL): StatPearls Publishing Copyright © 2024, StatPearls Publishing LLC. (2024)

[16] Mercuri E Seferian AM Servais L Deconinck N Stevenson H Ni X. Safety, tolerability and pharmacokinetics of eteplirsen in young boys aged 6−48 months with Duchenne muscular dystrophy amenable to exon 51 skipping. Neuromuscul Disord. (2023) 33(6):476–83. 10.1016/j.nmd.2023.03.008 37207382

[17] Mavrogeni S Markousis-Mavrogenis G Papavasiliou A Kolovou G. Cardiac involvement in Duchenne and Becker muscular dystrophy. World J Cardiol. (2015) 7(7):410–4. 10.4330/wjc.v7.i7.410 26225202 PMC4513493

[18] Batra A Barnard AM Lott DJ Willcocks RJ Forbes SC Chakraborty S. Longitudinal changes in cardiac function in Duchenne muscular dystrophy population as measured by magnetic resonance imaging. BMC Cardiovasc Disord. (2022) 22(1):260. 10.1186/s12872-022-02688-5 35681116 PMC9185987

[19] Mulder BJ van der Wall EE. Duchenne muscular dystrophy; a cardiomyopathy that can be prevented? Int J Cardiovasc Imaging. (2009) 25(1):65–7. 10.1007/s10554-008-9370-9 18830686

[20] Nigro G Comi LI Politano L Bain RJ. The incidence and evolution of cardiomyopathy in Duchenne muscular dystrophy. Int J Cardiol. (1990) 26(3):271–7. 10.1016/0167-5273(90)90082-g 2312196

[21] Kim HG Eun LY Park HK. Is it possible for children in Duchenne muscular dystrophy to preserve cardiac function with medical assistance? Children (Basel). (2020) 7(11):249. 10.3390/children7110249 33266491 PMC7700218

[22] Adorisio R Mencarelli E Cantarutti N Calvieri C Amato L Cicenia M. Duchenne dilated cardiomyopathy: cardiac management from prevention to advanced cardiovascular therapies. J Clin Med. (2020) 9(10):3186. 10.3390/jcm9103186 33019553 PMC7600130

[23] Łoboda A Dulak J. Muscle and cardiac therapeutic strategies for Duchenne muscular dystrophy: past, present, and future. Pharmacol Rep. (2020) 72(5):1227–63. 10.1007/s43440-020-00134-x 32691346 PMC7550322

[24] Kisel J Ballard E Suh ES Hart N Kapetanakis S Srivastava S. Cardioprotective medication in Duchenne muscular dystrophy: a single-centre cohort study. J Thorac Dis. (2023) 15(2):812–9. 10.21037/jtd-22-1528 36910051 PMC9992617

[25] Viollet L Thrush PT Flanigan KM Mendell JR Allen HD. Effects of angiotensin-converting enzyme inhibitors and/or beta blockers on the cardiomyopathy in Duchenne muscular dystrophy. Am J Cardiol. (2012) 110(1):98–102. 10.1016/j.amjcard.2012.02.064 22463839

[26] Rok M Wong TWY Maino E Ahmed A Yang G Hyatt E. Prevention of early-onset cardiomyopathy in Dmd exon 52−54 deletion mice by CRISPR-Cas9-mediated exon skipping. Mol Ther Methods Clin Dev. (2023) 30:246–58. 10.1016/j.omtm.2023.07.004 37545481 PMC10403712

[27] Iff J Desguerre I Liu Y Sarkozy F Tuttle E. Muntoni F. Association between exon-skipping therapy with eteplirsen and cardiac outcomes in Duchenne muscular dystrophy. J Neuromuscul Dis. (2026) 13(2):179–89. 10.1177/22143602251366721 40831143 PMC13141851

